# Exploring the experiences of parent caregivers of children with chronic medical complexity during pediatric intensive care unit hospitalization: an interpretive descriptive study

**DOI:** 10.1186/s12887-019-1634-0

**Published:** 2019-08-06

**Authors:** Janet E. Rennick, Isabelle St-Sauveur, Alyssa M. Knox, Margaret Ruddy

**Affiliations:** 1 The Montreal Children’s Hospital, McGill University Health Centre (MUHC), 1001 Decarie Boulevard, Montreal, Quebec H4A 3J1 Canada; 2 Ingram School of Nursing, Faculty of Medicine, McGill University, 680 Sherbrooke West, #1800, Montreal, Quebec H3A 2M7 Canada; 3 Centre for Outcomes Research and Evaluation, Research Institute of the McGill University Health Centre, 5252 de Maisonneuve West, 3F.47, Montreal, Quebec H4A 3S5 Canada

**Keywords:** Children with medical complexity, Complex care, Pediatric intensive care, Parents

## Abstract

**Background:**

Children with medical complexity (CMC) account for an increasing proportion of pediatric intensive care unit (PICU) admissions across North America. Their risk of unscheduled PICU admission is threefold compared to healthy children, and they are at higher risk of prolonged length of stay and PICU mortality. As a result of their sophisticated home care needs, parents typically develop significant expertise in managing their children’s symptoms and tending to their complex care needs at home. This can present unique challenges in the PICU, where staff may not take parents’ advanced expertise into account when caring for CMC. The study aimed to explore the experiences of parents of CMC during PICU admission.

**Methods:**

This interpretive descriptive study was performed in the PICU of one Canadian, quaternary care pediatric hospital. Semi-structured interviews were conducted with 17 parent caregivers of 14 CMC admitted over a 1-year period.

**Results:**

Parents of CMC expected to continue providing expert care during PICU admission, but felt their knowledge and expertise were not always recognized by staff. They emphasized the importance of parent-staff partnerships. Four themes were identified: (1) “We know our child best;” (2) When expertise collides; (3) Negotiating caregiving boundaries; and (4) The importance of being known. Results support the need for a PICU caregiving approach for CMC that recognizes parent expertise.

**Conclusions:**

Partnership between staff and parents is essential, particularly in the case of CMC, whose parents are themselves skilled caregivers. In addition to enhanced partnerships with health care professionals, needs expressed by parents of CMC during PICU hospitalization included improved communication with staff, and more attention to continuity of care in the PICU and across hospital services. Parent-staff partnerships must be informed by ongoing communication and negotiation of caregiving roles throughout the course of the child’s PICU hospitalization.

## Background

Recent decades have witnessed a dramatic decline in rates of infant and pediatric mortality and pediatric hospitalization [1–3], while chronic conditions requiring complex medical and nursing care account for a larger portion of admissions and days in hospital [4–6]. In the pediatric intensive care unit (PICU), advances in medical technology and postoperative care have allowed children with previously fatal conditions to survive. Many live with lifelong medical complexity, typically undergoing successive health crises and experiencing worsening health over time [7]. Children with medical complexity (CMC) have a threefold risk of unscheduled PICU admission compared to healthy children [4], and a higher risk of prolonged length of stay and PICU mortality [8]. It has been postulated that CMC and their parents constitute a distinct population in the PICU, facing different challenges than other critically ill children and their parents [9, 10].

CMC are children whose underlying medical conditions are expected to last longer than 1 year, are associated with high morbidity and mortality, and result in daily home care needs that are life sustaining and/or similar to care provided in hospital [11]. While diagnoses vary, these sophisticated care needs can include complicated medication regimens, assisted ventilation, oxygen support, tracheostomy care, enteral or parenteral nutrition, and central lines. Parents typically develop significant expertise in managing symptoms and tending to complex care needs at home [12]. Without close supervision, CMC are likely to deteriorate and require re-hospitalization, typically in the intensive care setting [8].

Yet, PICU staff may not take parents’ advanced expertise into account when caring for CMC [9, 13]. Exploratory studies of parents of CMC on hospital wards have found that while parents expect a collegial relationship with health care professionals [14–16], professionals often fail to acknowledge parents’ expertise [16, 17]. In fact, health care professionals’ assumptions regarding parental involvement in care may be based on their own (versus the parent’s) comfort level [12, 18]. One study explored the experiences of parents of children with severe antecedent disabilities (*n* = 8) admitted to a PICU [9]. Parents highlighted differences in their needs versus those of parents new to the unit, suggesting the PICU’s acute care model may not address the needs of parents familiar with both the critical care environment and the provision of high-tech care. These concerns are highlighted in calls for greater efforts to identify and address the needs of CMC and their families in the PICU [8, 10, 19].

In view of the growing prevalence of this population and new challenges regarding roles and relationships between parents and health care professionals, this study sought to elicit an in-depth understanding of parents’ experiences caring for CMC in the PICU. Findings will be used to enhance our understanding of how staff and parents can partner to care for this growing population of children who are no longer on the periphery of pediatric intensive care.

## Methods

The study received ethics approval from the hospital’s Research Ethics Board. Potential participants were informed of the study purpose, procedures, risks and benefits, and those who chose to participate provided written consent.

### Study design

Little is known regarding these parents’ perceptions of PICU hospitalization. We therefore used an inductive approach to capture the contextual and unique nature of each parent’s experience, while elucidating the shared realities of parents of CMC who become critically ill [20]. We specifically selected interpretive description, a qualitative design which was developed by a nurse researcher based on adaptation of traditional qualitative methods to the context of health experiences [21]. Interpretive descriptive studies aim to construct meaning within subjective experience and generate clinical practice implications in analysis of results. We worked from a clinically grounded question, and used open-ended interview questions to gain an in-depth understanding of parents’ experiences when their CMC were admitted to the PICU [20, 21].

### Setting and participants

Parents were recruited in the PICU of one Canadian, university-affiliated pediatric hospital. Interviews took place in a private room close to the PICU. Parents of all CMC admitted to the PICU over a 12-month period who met the following inclusion criteria were eligible to participate:

1. The child was followed by the hospital’s Complex Care Service.
2. The child had lived at home for at least 3 months prior to admission.
3. The parent was the child’s primary caregiver.
4. The child was admitted to the PICU for at least 3 days and deemed medically stable.
5. The parent read, wrote and spoke English or French.
6. The parent agreed to an audio-taped interview.

It was important that our sample reflect the diversity of diagnoses associated with the study population. As reasons for these children’s PICU admissions are often seasonal (e.g., respiratory illnesses peak in fall and winter), we approached all parents of CMC admitted over a 12-month period (2015–2016). This sampling strategy allowed us to capture information regarding parents’ experiences from a representative sample of the study population [21, 22].

### Data collection

Parents who met the study inclusion criteria were identified by the Nurse Manager (MR), and the staff nurse caring for the child asked the parent’s permission to be approached by a member of the research team (IS) who did not work in the PICU to explain the study. Parents who agreed were approached, and the study was explained. Those who agreed to participate were asked to sign a written consent form, and an interview was scheduled.

Child demographic and hospital baseline data, including level of illness severity (measured by the Pediatric Risk of Mortality Scale: PRISM III) [23] and number of tissue-damaging and non-tissue-damaging invasive procedures the child was exposed to (measured by the Invasive Procedure Score: IPS) [24] along with the child’s home care needs, were collected from the medical chart. Parent interview data was collected using semi-structured, open-ended interview questions (Table 1). Broad, open-ended questions were used to elicit parents’ unique reports of their PICU experience. Data collection and analysis occurred iteratively, with questions in later interviews reflecting emerging interpretation of content in earlier interviews. Interviews lasted between 1 and 2 h, and were audio-recorded to enhance rigor. Probing questions were used to promote elaboration of ideas and descriptions so that all possible responses could be elicited and clarified if necessary [12]. Parents were asked to provide demographic information about themselves and their family. Observational field notes described parents’ nonverbal responses, and any interruptions or distractions.

**Table 1:** Interview Questions

Interview Question	Probe(s)
Tell me about (child’s name) and his/her care needs at home	Who provides your child’s care at home?
	What is (child’s name) typical care routine?
Can you tell me why (child’s name) was admitted to the PICU?	
What has your experience been like since (child’s name) was admitted?	Is this a new experience for you?
	If no: Can you describe how this experience has differed for you?
As a parent (or caregiver), you are used to providing (child’s name) care at home. The staff in the PICU is now carrying out some of those caretaking needs, such as (name a home care task the parent discussed earlier). • How are you involved in your child’s care in the PICU?	Do you think that being involved/more involved in your child’s care is/would be helpful for you? For your child?
• How would you like to be involved?	
What can staff do to better support you while (child’s name) is in the PICU?	How can staff work together with you to care for your child? If the child was previously admitted to the PICU: Were there things staff did during your child’s previous admission(s) that you found helpful?
If a parent of a child with home care needs similar to (child’s name) was preparing for a PICU admission and asked you what to expect, and how to prepare – what would you tell that parent?	

### Data analysis

Parent and child demographic and hospital baseline information were analyzed using descriptive statistics. Audio recordings were transcribed verbatim and combined with field notes. Identifying information was removed from the transcripts and replaced with a study number, and transcript files were password-protected. Interview data were analyzed using the constant comparative method [25]. All investigators read the transcripts independently and conducted line-by-line coding describing key aspects of the transcript content [26]. Codes were discussed, and similar codes grouped into broader categories and compared within and across interviews to determine commonalities and variations [25, 27]. As interviews progressed, codes and categories were validated with participants to verify ongoing interpretation of the data, which also guided questions posed during subsequent interviews. Study team members continued to review the data until no new categories were generated. Through critical reflection, recurring experiences across categories were extracted to identify the final study themes [28]. The team agreed that data saturation had been achieved when new information produced little or no change in the data categories or themes [27, 29].

Study team members brought a variety of clinical and research perspectives to the analysis process, resulting in a deeper understanding of the interview findings. MR was the Nurse Manager of the PICU at the time of the study and brought an administrative perspective. ISS brought an in-depth understanding of the study population as an Advanced Practice Nurse in the Complex Care Service. She conducted interviews with all participants. JR approached the analysis as a senior nurse scientist with clinical and research background in pediatric critical care, and AK brought the perspective of a Research Assistant and a nurse with clinical experience in adult care. None of the study team members were involved in providing care to the children of any study participant.

While the diverse roles of study team members meant that they brought valuable perspectives to the data, steps were taken to ensure that codes, categories and themes closely reflected the participants’ responses. Researchers maintained an awareness of how their clinical and research experiences could influence their interpretation of the data. Several strategies contributed to the trustworthiness of the findings. Credibility and confirmability were enhanced through triangulation of multiple data sources (field notes, interview transcripts), and multiple team members to analyze and interpret the data. Interviewing parents of a diverse group of CMC and conducting member checks with participants enhanced credibility. The maintenance of a clear audit trail to ensure data could be traced back to its original source and outlining of all decisions made by the investigative team in coding and analyzing the data enhanced dependability and transferability.

## Results

Parents of 19 CMC who met the inclusion criteria were approached. The parent of one child refused, and four children were discharged before an interview could take place. A total of 17 parents of 14 children (79% of eligible admissions) were interviewed (Table 2). Eleven parents were interviewed individually, and parents of three children chose to be interviewed with their spouse. There were no striking differences between the content discussed by parents who were interviewed with their spouse and those who were interviewed alone. Participants’ children ranged in age from 10 months to 18 years, and had varying diagnoses and home care needs. They had varying levels of illness severity (PRISM III) [23] and were exposed to varying numbers of invasive procedures (IPS) [24] (Tables 2 and 3). Eight of the 14 families reported receiving some paid support in the home (either publically or privately funded), ranging from half a day of support with household tasks per week through 8.5 h of nursing care every night for one child, who was on a ventilator. All parents were expert caregivers, and described challenges reconciling their needs, expectations, and knowledge with staff expectations and the PICU culture of care. One parent explained: “[Our children] don’t fall under [usual PICU care] protocols. Its protocol plus.”

**Table 2:** Parent and Child Demographics & Hospital Baseline Data

	n (%)	Median (Range)
Parent (*n* = 17)		
Age (years)		40 (23–54)
Relationship to child (mother)	10 (59%)	
Marital Status		
Single	1 (6%)	
Separated (living together)	1 (6%)	
Married/domestic partnership	15 (88%)	
Number of people in the home		4 (3–10)
One or more parents work outside the home (*n* = 14)	11 (79%)	
Child (*n* = 14)		
Sex (female)	7 (50%)	
Age (years)		4.5 (0.83–18)
Length of Stay (days)		
PICU		10 (1–76)
Hospitalization		11.5 (8–230)
IPS^a^		326.5 (130–1852)
PRISM-III^b^		4 (0–10)
Previous PICU hospitalizations		5.5 (1–15)
Admitting Diagnostic Category^c^:		
Cardiovascular	1 (7%)	
Respiratory	10 (71%)	
Neurological	2 (14%)	
Infectious Diseases	1 (7%)	
Gastro-Intestinal	1 (7%)	
Orthopedics	1 (7%)	
Chronic Conditions^c^:		
Respiratory Disease	2 (14%)	
Neurological & Neuromuscular Diseases	10 (71%)	
Gastro-Intestinal Disease	6 (43%)	
Orthopedic Disorder	5 (36%)	
Renal Disease	1 (7%)	
Congenital Disorder	3 (21%)	

^a^IPS: Invasive Procedure Score [29]

^b^PRISM-III: Pediatric Risk of Mortality Score, version 3 [28]

^c^Some children had multiple admitting diagnoses and/or chronic conditions

**Table 3:** Child Home Care Needs

	n (%)
Assistance in Activities of Daily Living (ADLs)^a^	14 (100%)
Medication Administration	14 (100%)
Central Vascular Access Device Care	3 (21%)
Respiratory Care:	
Ventilatory Assistance (Invasive; Non-invasive)	9 (64%)
Tracheostomy Care	3 (21%)
Aspiration of Secretions (oral; naso-pharyngeal; tracheal)	12 (85%)
Cough Assist Techniques (inspiratory; expiratory; inspiratory/expiratory)	7 (50%)
Oxygen Administration & Oxygen Saturation Monitoring	9 (64%)
Diaphragmatic Pacer	1 (7%)
Nutrition & Hydration	
Enteral Nutrition Care	10 (71%)
Parenteral Nutrition Care	2 (14%)
Intravenous Hydration	1 (7%)
Ostomy Care (e.g. colostomy; ileostomy)	2 (14%)
Physiotherapy exercises	12 (85%)

^a^ADLs include feeding, bathing, positioning, transfers, installing/removing orthotics, etc

Findings revealed the need for a different approach to PICU care for CMC, with an emphasis on establishing parent-staff partnerships to optimize patient care. Four major themes were identified (Table 4): (1) “We know our child best,” which included subthemes a) Living with uncertainty and b) “Hospital care needs are similar to home care needs;” (2) “When expertise collides,” which included differences of opinion or breakdowns in communication between a) parents and health care professionals, and b) health care professionals; (3) “Negotiating caregiving boundaries;” and (4) The importance of being known.

**Table 4:** Themes

Theme	Subthemes	Quotations
“We know our child best”		*“They’re the medical professionals, and we’re the professionals of our child.”*
	*Living with uncertainty*	*“…living life one day at a time. You can never think about what’s going to happen next.”*
	*Hospital care needs are similar to home care needs*	*“If she’s well enough, I’m going to bring her home... If not, she stays [in the PICU]. I can’t leave her [on the ward], where there’s less observation than if I were watching her.”*
When expertise collides	*Parents and health care professionals*	*“It’s like, ‘I’m the health care professional, you’re just a parent.’ I might not have their professional training… but I do this every day, 2–3 times per day with my child.”*
	*Between health care professionals*	*“…a doctor often won’t ask for help from another doctor”*
Negotiating caregiving boundaries		*“…there’s a confidence that needs to be established. When the nurse sees you do the right things at the right times, she is more inclined to let you go”*
The importance of being known		*“As [staff] got to know us, they saw that we know [our child] very well… so they came to look for us when they were ready to talk about her [in rounds]”*

### “We Know Our Child Best”

Parents provided complex, continuous care at home and developed expert knowledge regarding their child’s health care needs. Their intimate understanding of their child’s communication, along with their medical assessment and caregiving expertise, were considered central to their child’s quality of care at home and in hospital. While some children were able to communicate independently or with assistive technology, others were not. Several parents described specific, unique physical cues that helped them understand their child’s level of comfort and care needs. One parent explained, “you need to know his way of communication and how he responds to things… to be able to assess my son properly and fairly.”

Parents’ expert knowledge of their child’s health included their medical history, current condition, and unique responses to caregiving interventions. The need for continuous caregiving at home, including sophisticated medical interventions, presented an extreme demand on parents. One mother reflected:

…we normalize things. The care we give is extreme. I tell myself it’s nothing because I know worse. You see children who have even more needs, and then you say to yourself this is nothing. But it’s not nothing. It’s enormous.

Parents also brought knowledge of their child’s past hospitalization experiences to the current PICU admission. For example, one father expressed concern that plans for his son’s upcoming discharge from the PICU might be premature: “by you pushing him out the door a day earlier, if he’s not ready we’re gonna be back here in 2 days.” Parents attempted to use their past experiences to guide current care practices.

#### Living with uncertainty

Parents’ narratives revealed emotional and psychological challenges associated with caring for CMC. In the face of uncertain illness trajectories, parents constantly weighed the risks of their decisions. One parent stated “there are a lot of grey areas with kids like [him].” Another stressed the importance of “living life one day at a time. You can never think about what’s going to happen next.” Uncertainty was associated with the life-limiting nature of many children’s conditions. For two parents, making decisions about care during resuscitation was particularly difficult given their child’s uncertain future. Uncertainty was also associated with children’s fragility; a child who was fine one moment might change rapidly in the next moment. One father explained “It’s quite difficult to get a routine for her in place, to say that yes, today at 2 o’clock we aim to go to the shops and she could be having seizures.” Parents were confronted with the continual need to assess their child’s changing health situation to make decisions in his/her best interest.

#### Hospital care needs are similar to home care needs

At home, parents provided sophisticated care to CMC (Table 3). One mother described that care as more intensive than care her daughter might receive on the hospital ward:

“I can’t leave her [on the medical unit], where there’s less observation than if I were watching her… For her to have less care [in hospital] than when I care for her [at home], it’s not normal.”

Parents were capable of responding to deterioration in their child’s condition at home. Several parents described changing the level of care they provided before deciding to go to hospital: “the few days before we decide we have to go to [hospital] are demanding, because we start the ‘acute care protocol,’ that’s what we call it, at home.” Parents continued to provide sophisticated care in the PICU: “We do exactly the same things here [as at home].” Most understood their knowledge as complementary to that of health care professionals: “I’ve seen her progression; I’ve seen where we’re at and how it is now. I don’t know what we need to do now that we’re here, they know better than me how to care for her… I will give them the state of the situation.” As one parent stated, “They’re the medical professionals, and we’re the professionals of our child.”

### When expertise collides

#### Parents and health care professionals

Parents, PICU staff, and health care professionals from other hospital services contributed unique knowledge and skills to the care of CMC; however, integrating these diverse contributions was challenging. Interactions could improve care or could result in communication challenges and conflicts.

All parents felt the need to be vigilant while their child was hospitalized: “I always feel responsible to supervise.” Another parent noted, “Humans make mistakes. So we have to double check.” Parents were vigilant about their child’s comfort, noting the importance of reminding staff of the child’s unique sensitivities and needs. Most spent considerable time at the bedside, sometimes having a family caregiver present at all times.

While they felt they played an important role, parents did not always feel welcome. “I feel as if they’re looking at it like we’re taking their job away or they’re annoyed by us being there… We’re his parents, we’re adding an extra hand.” Parents felt their expertise was not always acknowledged: “It’s often, ‘I’m the health care professional, you’re just a parent.’... I might not have professional training to do this, but I do it every day, 2-3 times per day with this child.” Parents struggled when physicians made decisions without consulting them, when information they provided about their child’s preferences and needs was not acknowledged, or when the team did not apprise them of changes in the child’s care plan. Parents were very appreciative when their contributions to care were acknowledged. One father noted “Everybody has their own way, but very close to 100% [of staff] takes our suggestions and comments and goes with them.”

#### Between health care professionals

Communication challenges between health care professionals had an impact on parents. All families had long-term relationships with staff from Complex Care and other hospital services, and parents became frustrated when the knowledge and decision-making advice of those providers was not integrated into their child’s PICU care. “I don’t think they respect [Complex Care] as much as they should… and that’s disappointing because Complex Care knows my son much better than they do.” Another parent described advocating for his daughter across services, because, “…a doctor often won’t ask for help from another doctor.”

One parent described a positive communication experience: “We met with Complex Care and the [PICU] team. I find it’s good for all of us to be on the same page at the beginning.” Parents valued teamwork between the PICU and other health services. “[Complex Care staff] know what [our daughter] looks like healthy… I think if they work more together, it’ll give us a sense of trust [and] comfort.”

Within the PICU, parents found that regular team changes impacted continuity of care. When nurses applied rules differently, parents who were already stressed found it “unnerving.” One parent pointed out “…the residents change often, maybe they need to take more time to look at the medical history of each patient.”

### Negotiating caregiving boundaries

Collaboration between parents and health care professionals took time and work to establish. Parents described a spectrum of involvement in PICU care, from doing as much of their child’s care as possible to taking as much respite time as possible. All parents wanted their presence and involvement to be welcomed, and to feel part of the care team. One mother said she felt supported “…when they include us in their discussions and take our ideas into consideration.” Some parents felt their participation improved communication as they provided continuity. One father tried to be present for rounds to address gaps in knowledge about his child or miscommunications between health care professionals. Feeling listened to was critical, and fostered trust in health care professionals. “I really like being asked ‘How do you do it at home? How does it work?’ It shows an openness… to open the door and go, okay, I’m listening to you and I’m going to take what you say into consideration…”.

It was often challenging for parents to establish their role as caregivers in the PICU. Some wanted a specific level of involvement: “…it was a fight, to get our boundaries of what we wanted and what they were willing to let us do.” Others adapted to health care professionals’ boundaries: “…I would not do it, if they don’t agree. We have to work together.” Many parents had developed positive working relationships with some PICU team members but had to constantly renegotiate their role at shift change. Parents relied on staff they knew to advocate for their involvement in care. “When a staff tells another staff that the parents know what they’re doing, it’s more readily accepted.” Parents felt they needed to prove their competency. “I think there’s a confidence to establish… when the nurse sees that you are logical in what you do, and you do the right things at the right times, she is more inclined to let you go.” Parents also assessed health care professionals’ competency. One mother said: “Sometimes I’ll say ‘ok you do it, and after I’ll check it’,” explaining that she needed to feel confident in the staff’s ability to suction her daughter before taking respite.

Accepting that staff might not care for their child exactly as they would was challenging for some parents. Yet it was equally challenging to be constantly present. Parents expressed a need for respite during their child’s hospitalization: “[At home] it’s demanding. Once things have stabilized, and we’re admitted and all of that, it’s important to sit, to try and trust, and… take some respite.”

### Importance of being known

Parents felt secure and comfortable when their family was known by PICU staff. Staff who knew the family were familiar with the child’s care routines, and understood parenting styles, allowing them to build rapport with parents and children. Relationships with staff often developed over repeated admissions. “For sure now they know him better, so they know a little more about the care routines, our way of doing it…. their care is more individualized.” Some parents had arranged for their child to have a primary nurse; but even those who did not identified particular staff who knew their child well, and whom they trusted. One mother explained “the third day [with the same nurse, my child] is at ease, he doesn’t complain anymore.”

Parents appreciated the primary nurse’s ability to interpret their child’s responses and felt that having the same nurse during a subsequent admission improved their PICU experience. One parent recounted a time when a respiratory therapist who knew the child well anticipated deterioration in her condition and ensured that help arrived in time: “When it’s people who have known her for a long time, they know what could happen.” Parents reported that family meetings were better experiences when their child’s primary nurse was present. They valued the primary nurse as a person to provide support during and following difficult conversations regarding their child’s ongoing care (e.g., conversations about resuscitation plans).

As the team got to know them, parents felt they were included more often in rounds and care planning discussions. “[Staff] saw that we know her very well… so they came to look for us when they were ready to talk about her.” As parents developed confidence in health care professionals, they felt more able to take a break. One parent explained: “…we know certain nurses [are] more gentle, and they’ll be more like us, so we’ll let them do a bit more, and we’ll be able to go and eat.” Parents felt welcomed and supported when they had developed relationships with staff: “we come to have confidence in people because we know them better. It’s like a family.” However, some parents felt that when the PICU team knew they would be present, their child received less attention or was assigned to a nurse with a second patient.

## Discussion

Parents developed expert knowledge regarding their child’s health care needs by providing continuous, complex care at home. Most understood their knowledge as complementary to that of health care professionals, however integrating diverse contributions to care could be challenging. Collaboration took time and constant work to establish. Negotiating parent involvement in their child’s care and establishing caregiver partnerships was not always supported. Parents valued PICU staff who took the time to get to know their child and family well. Ultimately, parents in this study considered partnerships with PICU staff to be central to the provision of excellent care for their children.

Parents in our study were expert care providers and expressed a desire to be recognized as such and involved in care, including care planning and technical procedures. While some parents reported successfully negotiating their desired level of involvement, others felt excluded or, alternatively, that they were relied upon to provide bedside care, thus limiting their ability to take much-needed time for respite. Parents needed PICU staff to be attentive to their desired level of involvement in care on an ongoing basis. Their needs for support and readiness to contribute to their child’s care could change over the course of their child’s hospitalization, and they needed staff to be ready to engage in ongoing negotiation and to react flexibly to their changing needs. Positive experiences of recognition and involvement were considered inconsistent across PICU staff; yet, parent-staff partnerships are an important component of patient and family centered care (PFCC), an approach with clear health benefits for CMC and their families [13, 30, 31]. A prospective ethnography identified a divergence between one PICU’s stated value of PFCC, and daily practice patterns which presented barriers to parent involvement [32]. The presence of a gap between PICU staff and family perceptions of “what families want and need” was identified. Our results support this finding.

Creating and maintaining partnerships with expert parents may not be intuitive to PICU staff. In our previous work, we found that nurses felt unprepared to partner with parents of CMC and expressed a need for further education to facilitate the development of effective caregiver relationships [33]. Suggested methods of providing support for parents of CMC in the community, including openly acknowledging parent expertise and providing information and reassurance, may be transferable to the PICU [34]. Improving nurses’ and other health care professionals’ preparation to incorporate parent expertise into PICU care routines could be beneficial not only for CMC and their parents, but also for health care professionals who can find this population challenging to work with. Given that both PICU staff and parents of CMC report challenges establishing productive working relationships during PICU hospitalization, both parties may benefit from further preparation around the establishment of caregiving partnerships. One strategy could be to provide education to health care professionals about the perspectives and experiences of parents and CMC during PICU hospitalization.

In the case of critically ill CMC, several authors have advocated for adjustments to the traditional approach to PICU care, which aims to rescue the critically ill child and return them to their previously healthy baseline [10, 35]. One group conducted semi-structured interviews with 44 staff and seven parents exploring their perspectives on ICU care for children with chronic critical illness (defined as technology dependence and recurrent or prolonged ICU admissions) [36]. Data were content analyzed and similar challenges to those identified by parents in our study were reported, such as constantly changing clinical teams and communication difficulties between services and between staff and families. Their findings were based primarily on staff perspectives, and this is reflected in important areas where our study findings diverge. Of particular note, while staff expressed moral distress regarding conflicts with parents about the ethics of continuing interventionist care for CMC or children with chronic critical illness [33, 36–38], parents in our study did not refer to this as a source of conflict. Rather, two parents in our study stressed the importance of receiving support from staff who knew their family well during conversations about continuing care. Differences in how parents and staff approach and understand discussions about the goals of continuing intensive care appear to warrant further research. Our findings highlight the importance of incorporating parents’ perspectives into PICU practice changes, as families’ perspectives and needs may differ from those of staff.

Parents of CMC face multiple challenges related to their child’s health and care needs [39–41]. These are exacerbated during PICU stays, as parents must adapt to the PICU environment, manage complex decision-making, and balance other home and work responsibilities. A recent study reported that parents of CMC sought hospital care only when they were no longer comfortable at home and concluded that improving parents’ self-efficacy in caring for their child at home could reduce the number of hospitalizations [42]. Yet parents in our study expressed considerable confidence in caring for their children, whether at home or in hospital, suggesting that for these families, improving the hospitalization experience might be a more appropriate goal than reducing the number of hospitalizations. Similarly, another study aimed to increase the proportion of CMC discharged from an acute care medical unit within the first 2 hours of meeting medical discharge goals by proactively addressing their home care needs [43]; yet, parents in our study expressed concern that accelerated discharge from the PICU could result in unmet care needs for their children. Our finding was supported in a study of CMC families’ priorities for hospital-to-home transition, in which parents expressed the importance of not feeling “rushed out the door” [44]. The divergence between recently published interventions to improve hospital experiences for CMC and their families and the needs reported by participants in our study presents an important implication for future research. Our results suggest there is a need to consult families as early as possible in developing any clinical practice changes or interventions intended to benefit them. To effectively improve the hospital experiences of CMC and their families, the caregivers who know them best – their parents – should be involved in the design and implementation of such changes. We recommend that future studies aiming to improve care for this population include parents as collaborators from study design to execution of interventions.

An integrative review examining interactions between parents of technology-dependent children and health care professionals in the home found that parents felt responsible for their child’s safety, wanted their caregiving expertise to be recognized, experienced communication challenges and conflicts with health care professionals, and appreciated continuity of care [45]. The parents in our study expressed similar needs during PICU hospitalization. They emphasized their need to be known and to have their own and their child’s primary care providers’ (e.g., the Complex Care Service) knowledge of their child integrated into PICU care. They expressed the need for better continuity between hospital care services. Ensuring that inter-professional communication is maximized during PICU hospitalization would help to address that need.

### Limitations

This study took place in one quaternary care, university-affiliated pediatric center. This may limit the transferability of findings. To address this, a detailed description of the study setting and patient population have been provided. In addition, parents were interviewed during their child’s PICU stay, and while this means that they were able to report their experience with immediacy, it was also a stressful time for them. Some parents may have been reluctant to disclose concerns while their child was actively undergoing care in the PICU. However, all children were medically stable at the time of recruitment, the interviewer was not a member of the PICU staff, and parents were forthcoming and reported both positive and negative aspects of their PICU experiences.

## Conclusion

Partnership between staff and parents is essential, particularly in the case of CMC, whose parents are themselves skilled caregivers. Recent reports of interventions to improve care for hospitalized CMC and their families target priorities that differ from those expressed by parents in our study. We found that the needs expressed by parents of CMC during PICU hospitalization included enhanced partnerships with health care professionals, improved communication with staff, and more attention to continuity of care in the PICU and across hospital services. Parents expressed a need for more systematic incorporation of their caregiver expertise into care. Parent-staff partnerships must be informed by ongoing communication and negotiation of caregiving roles throughout the course of the child’s PICU hospitalization.

## Data Availability

The datasets generated and analysed during the current study are not publicly available given that complete interview data are confidential. Representative quotes are included in this published article.

## Abbreviations

CMC: Children with Medical Complexity
PFCC: Patient and Family-Centered Care
PICU: Pediatric Intensive Care Unit
